# The Value of BISAP Score for Predicting Mortality and Severity in Acute Pancreatitis: A Systematic Review and Meta-Analysis

**DOI:** 10.1371/journal.pone.0130412

**Published:** 2015-06-19

**Authors:** Wei Gao, Hong-Xia Yang, Cheng-En Ma

**Affiliations:** Department of Intensive Care Unit, the Second Affiliated Hospital of Shandong University, Jinan, 250033, China; University of Valencia, SPAIN

## Abstract

**Purpose:**

The Bedside Index for Severity in Acute Pancreatitis (BISAP) score has been developed to identify patients at high risk for mortality or severe disease early during the course of acute pancreatitis. We aimed to undertake a meta-analysis to quantify the accuracy of BISAP score for predicting mortality and severe acute pancreatitis (SAP).

**Materials and Methods:**

We searched the databases of Pubmed, Embase, and the Cochrane Library to identify studies using the BISAP score to predict mortality or SAP. The pooled sensitivity, specificity, likelihood ratios, and diagnostic odds ratio (DOR) were calculated from each study and were compared with the traditional scoring systems.

**Results:**

Twelve cohorts from 10 studies were included. The overall sensitivity of a BISAP score of ≥3 for mortality was 56% (95% CI, 53%-60%), with a specificity of 91% (95% CI, 90%-91%). The positive and negative likelihood ratios were 5.65 (95% CI, 4.23-7.55) and 0.48 (95% CI, 0.41-0.56), respectively. Regarding the outcome of SAP, the pooled sensitivity was 51% (43%-60%), and the specificity was 91% (89%-92%). The pooled positive and negative likelihood ratios were 7.23 (4.21-12.42) and 0.56 (0.44-0.71), respectively. Compared with BISAP score, the Ranson criteria and APACHEⅡscore showed higher sensitivity and lower specificity for both outcomes.

**Conclusions:**

The BISAP score was a reliable tool to identify AP patients at high risk for unfavorable outcomes. Compared with the Ranson criteria and APACHEⅡscore, BISAP score outperformed in specificity, but having a suboptimal sensitivity for mortality as well as SAP.

## Introduction

Acute pancreatitis (AP) is the most frequent gastrointestinal cause of hospitalization in the United States, with an annual cost of over 2.5 billion dollars [1,2]. The prognosis of AP depends on its severity, which was classified as mild, moderate, or severe by the latest revised Atlanta classification [3]. Most patients present with mild or moderate AP, and only 15–20% of patients have severe AP (SAP) [4]. Notably, the mortality of mild or moderate AP is far less than that of SAP. The mortality is approximately 1% among all AP patients, but reaching as high as 20% to 30% among those with severe course [5].

It is of clinical significance to identify the patients most likely to develop SAP after admission, which will assist triage and the initiation of aggressive early treatment [3]. A series of severity scoring systems have been developed for the early detection of SAP. Currently, the Ranson criteria and the Acute Physiology and Chronic Health Examination (APACHE)IIsystem are most widely used in clinical practice [6,7]. However, they are very cumbersome and complex for quick evaluation. In 2008, the Bedside Index for Severity in Acute Pancreatitis (BISAP) score was proposed for the early recognition of patients at risk of mortality. This 5-point scoring system is comprised of five variables: blood urea nitrogen level > 25 mg/dl, impaired mental status, development of systemic inflammatory response syndrome (SIRS), age > 60 years, and presence of pleural effusion [8,9]. Compared with traditional scoring systems, BISAP is more convenient to use with fewer items. Several studies have been conducted to validate the BISAP score. However, they differed in many aspects, such as population, cutoffs, and clinical endpoints, which result in a broad range of predictive accuracy. Thus, we performed this systematic review and meta-analysis to quantify the accuracy of BISAP score for predicting mortality and severity of patients with AP. We also compared the BISAP score with the traditional scoring systems.

## Methods

### Search Strategy

The overview of the meta-analysis was conducted in accordance with the Preferred Reporting Items for Systematic Reviews and Meta-analysis (PRISMA) statement [10]. We selected all relevant articles published between 1950 to December 2014 by searching Pubmed, Embase and the Cochran Library. Medical subject heading terms used in the search included “acute pancreatitis”, “pancreatic necrosis”, “necrotizing pancreatitis”, “bedside index” and “BISAP”. The language was limited to English. We also manually searched conference proceedings and the references of selective articles to identify additional potentially relevant studies.

### Selection Criteria

The inclusion criteria for the meta-analyses were as follows: (1) studies were published in peer-reviewed, English-language journals from January 1980 to December 2014, and conference abstracts were only included when they provided adequate relevant information for assessment; (2) the BISAP score was used for the prediction of mortality or severity in patients with AP; (3) sufficient data on clinical outcomes were available for the calculation of the test performance (sensitivity, specificity, and diagnostic OR).

### Data Extraction and Quality Assessment

Two independent reviewers (WG and HXY) screened the titles and abstracts. Studies that satisfied the selection criteria were retrieved for fulltext evaluation. Any discrepancy was resolved by consensus or by consulting a third author (CEM). The following data were extracted from each included study in standardized forms: first author’s name, publication year, study design, location, sample size, mean age, main etiology, male percentage, cut-off value, clinical endpoints, prevalence of SAP, defined criteria of SAP, and study period. The raw data were summarized by 2×2 contingency tables of BISAP score against clinical outcomes.

No single quality assessment tool has been developed to appraise the methodological quality of studies of predictive score systems. Based on consensus among authors, we applied a revised 7-item assessment tool [11], which was derived from the widely used Newcastle-Ottawa Scale (NOS) and QUADS tool. The following seven criteria were used for quality assessment: patients selected in an unbiased fashion (consecutive or random sample); study sample representative of a wide spectrum of the severity of AP; predictor variables assessed without knowledge of the outcome; outcome assessed without knowledge of the predictor variables; outcomes accurately defined (especially SAP); the clinical data available when interpreting the BISAP score were the same as those available in practice; adequacy of follow-up (follow-up rate > 90%) (S1 Table).

### Definition of Outcomes

Previously, SAP was defined as organ failure and/or local complications by the 1992 Atlanta criteria [4]. In 2012, the revised Atlanta classification differentiated organ failure into transient and persistent. Transient organ failure is organ failure that is present for <48 h. Persistent organ failure is defined as organ failure that persists for >48 h. SAP was defined as persistent organ failure (POF) [3]. Organ failure involved the respiratory, cardiovascular and renal systems, and was defined as a score of 2 or more for one of these three organ systems using the modified Marshall scoring system [3]. Conforming to the latest consensus, we selected in-hospital mortality and SAP of 2012 Atlanta criteria, namely POF, as our primary clinical outcomes.

### Statistical Analysis

The statistical software Meta-Disc (version 1.4; Clinical Biostatistics, Ramony Cajal Hospital, Madrid, Spain) was used for meta-analyses [12]. We compared a total BISAP score of ≥3 with a score of <3. Additionally, sensitivity analysis was conducted for the cut-off of ≥2. Results were obtained by direct extraction or by indirect calculation. Pooled summary statistics with 95% confidential intervals (CIs) of sensitivity, specificity, positive likelihood ratio (PLR), negative likelihood ratio (NLR) and diagnostic OR (DOR) for clinical outcomes were calculated from each study. The random-effects model of DerSimonian and Laird was used for pooling the results [13]. A PLR higher than 5 and a NLR below 0.2 provide strong diagnostic evidence [14]. Further, the summary receiver operating characteristic (SROC) curve was generated and expressed by the Q*index and area under the curve (AUC). The threshold effect was indicated when a "shoulder arm" pattern was shown by the SROC curve, or when the Spearman correlation coefficient in the threshold analysis showing a strong positive correlation. The likelihood ratios, DORs, and SROC curves are more valuable for evaluating the diagnostic accuracy than sensitivity or specificity, as they consider both the sensitivity and specificity data. We used the Cochran’s Q test and I^2^ statistic to quantify the statistical heterogeneity between studies. A P value of less than 0.05 by Cochran’s test, and an I^2^ statistic greater than 50% suggested substantial heterogeneity [15]. The publication bias of included studies was assessed visually by funnel plot and statistically detected by Deek’s test [16], which were conducted using the STATA software (version 12.0; Stata Corporation, College Station, Texas). We inferred several potential sources of heterogeneity *a priori*: (1) study design (prospective or retrospective); (2) sample size (< 300 or ≥ 300); (3) cut-off (2 or 3); (4) main etiology of AP (biliary stone or alcohol); (5) prevalence of SAP (< 10% or ≥10%). Subgroup analyses and univariate meta-regression analyses were conducted to explore heterogeneity. A P-value of < 0.1 was considered significant for the examination of publication bias or heterogeneity.

Inter-rater reliabilities were calculated by the Cohen κ statistics with 5 levels of agreement, namely poor (κ = 0.00–0.20), fair (κ = 0.21–0.40), moderate (κ = 0.41–0.60), good (κ = 0.61–0.80), and very good (κ = 0.81–1.00) [17].

## Results

### Literature Search

**Fig 1:**
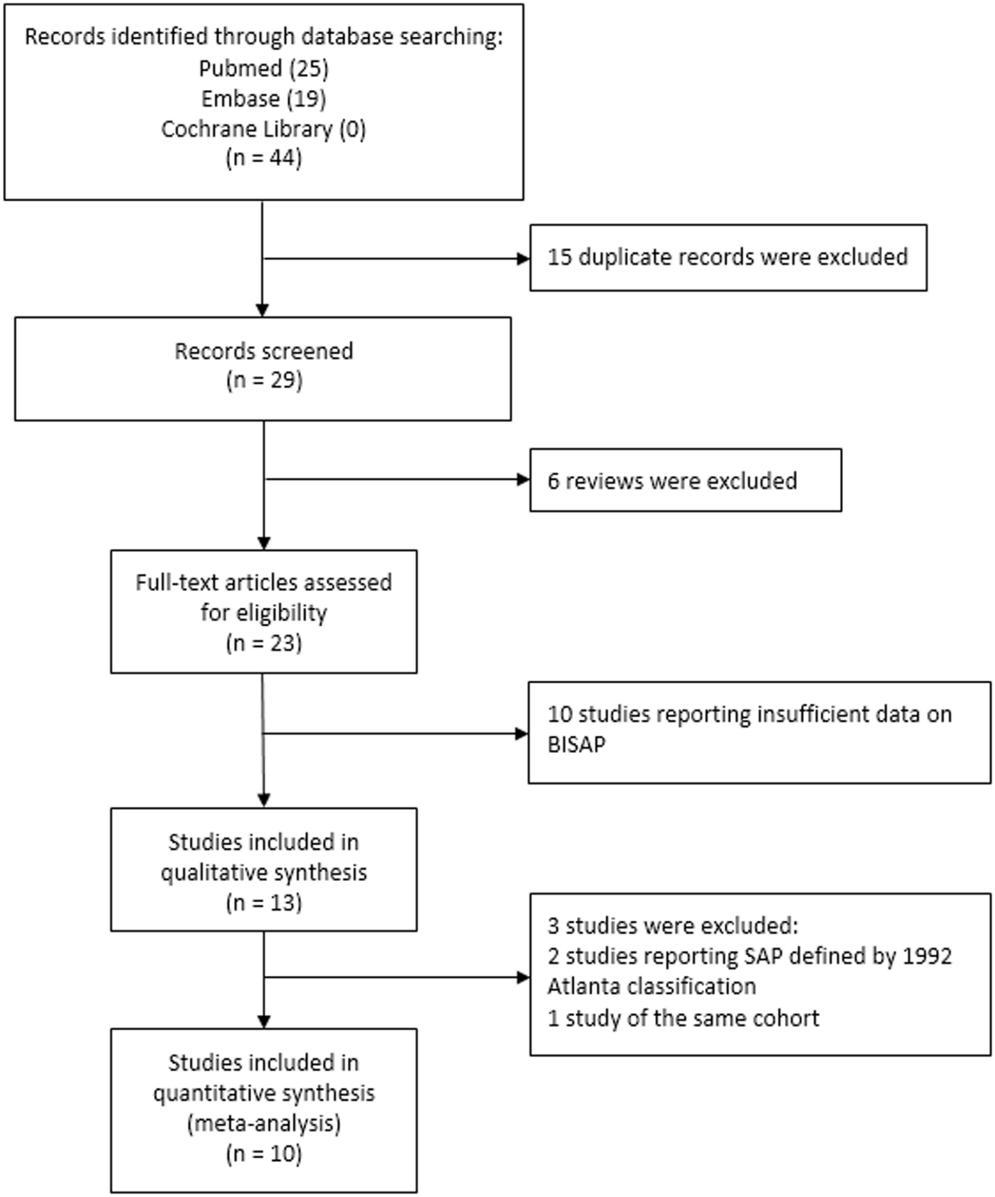
The flowdiagram for study selection process.

Fig 1 showed the selection process of eligible studies. Our initial search identified 44 records, including 25 records from Pubmed and 19 records from Embase. After removing 15 duplicate records and 6 reviews, 23 studies remained for assessment. Ten studies were excluded due to insufficient data to calculate the effect estimates, leaving thirteen studies included into the qualitative synthesis. Further, two records were excluded as they studied SAP defined by the 1992 Atlanta classification [18,19]. Two studies investigated the same cohort [20,21], and the study with more comprehensive data was selected [21]. Finally, 10 studies were included into meta-analyses. The manual search of reference lists of these articles did not produce any new eligible record. Agreement on selection of studies between two assessors was very good (κ = 0.91).

### Study Characteristics

Ten studies were eligible for meta-analyses (Table 1). As two studies had both derivation and validation cohorts [9,22], 6 retrospective cohorts [9,23–26], and 6 prospective cohorts were identified [21,22,27–29]. The 12 cohorts enrolled 38985 patients with AP from 4 countries. Six cohorts were conducted in the United States [9,21,22,28], two in Korea [23,24], two in China [25,26], and two in India [27,29]. The mean age ranged from 42 to 54 years. The proportion of males ranged from 49 to 71. Six studies had a cut-off of 3 [9,21,23,26,28,29], three studies of 2 [22,24,27], and one studies of both 2 and 3 [25]. The prevalence of SAP ranged from 5% to 43%. All studies calculated the BISAP score within 24 hours after admission.

**Table 1:** Characteristics of included studies for meta-analysis.

Author (year)	Location	Study design	Sample size	Mean age, y	Main etiology	Male, %	Cut-off score	Endpoints	SAP, %	Definition of SAP	Study period
Wu (2008)	USA	Retrospective	17922	Median: 53	Biliary	51	3	Mortality	NA	NA	2000–2001
	USA	Retrospective	18256	Median: 53	Biliary	49	3	Mortality	NA	NA	2004–2005
Singh (2008)	USA	Prospective	397	52	Biliary	49	3	Mortality, OF, persistent OF, PNes	5	2012 criteria	2005–2007
Papachristou (2010)	USA	Prospective	185	52	Biliary	51	3	Mortality, SAP, PNes	22	2012 criteria	2003–2007
Mounzer (2012)	USA	Prospective	256	Median: 51	Biliary	52	2	Persistent OF	24	2012 criteria	2003–2010
	USA	Prospective	397	Median: 52	Biliary	49	2	Persistent OF	9	2012 criteria	2005–2007
Chen (2013)	China	Retrospective	497	54	Biliary	55	2, 3	Mortality, SAP, OF, PNec	20	1992 criteria	2005–2010
Cho (2013)	Korea	Retrospective	299	52	Alcohol	70	3	SAP, mortality	7	2012 criteria	2008–2010
Khanna (2013)	India	Prospective	72	41	Biliary	51	2	SAP, PNes, mortality	43	1992 criteria	2010–2012
Park (2013)	Korea	Retrospective	303	52	Alcohol	71	2	Mortality, SAP, PNes, OF	10	1992 criteria	2007–2010
Senapati (2014)	India	Prospective	246	42	Alcohol	62	3	Mortality, OF, persistent OF, PNes,	7	2012 criteria	2011–2013
Zhang (2014)	China	Retrospective	155	52	Alcohol	59	3	Mortality, SAP, PNes	17	2012 criteria	2010–2013

OF, organ failure; PNes, pancreatic necrosis; SAP, severe acute pancreatitis.

### Quality Assessment

The inter-observer agreement of the quality assessment for the 10 studies was 93% with a κ value of 0.86. All studies enrolled patients in an unbiased fashion, with a wide spectrum of severity. The BISAP score was assessed blinded to outcome in 5 (50%) studies. No study clearly reported that the assessment of outcomes was blinded to the BISAP score. Generally, definitions of clinical outcomes were standard and followed the international Atlanta consensus. In all studies, the clinical data available when interpreting the BISAP score were the same as those available in practice. Patients were followed-up adequately in all studies. (S1 Table)

### Results of BISAP Score

#### Mortality

**Fig 2:**
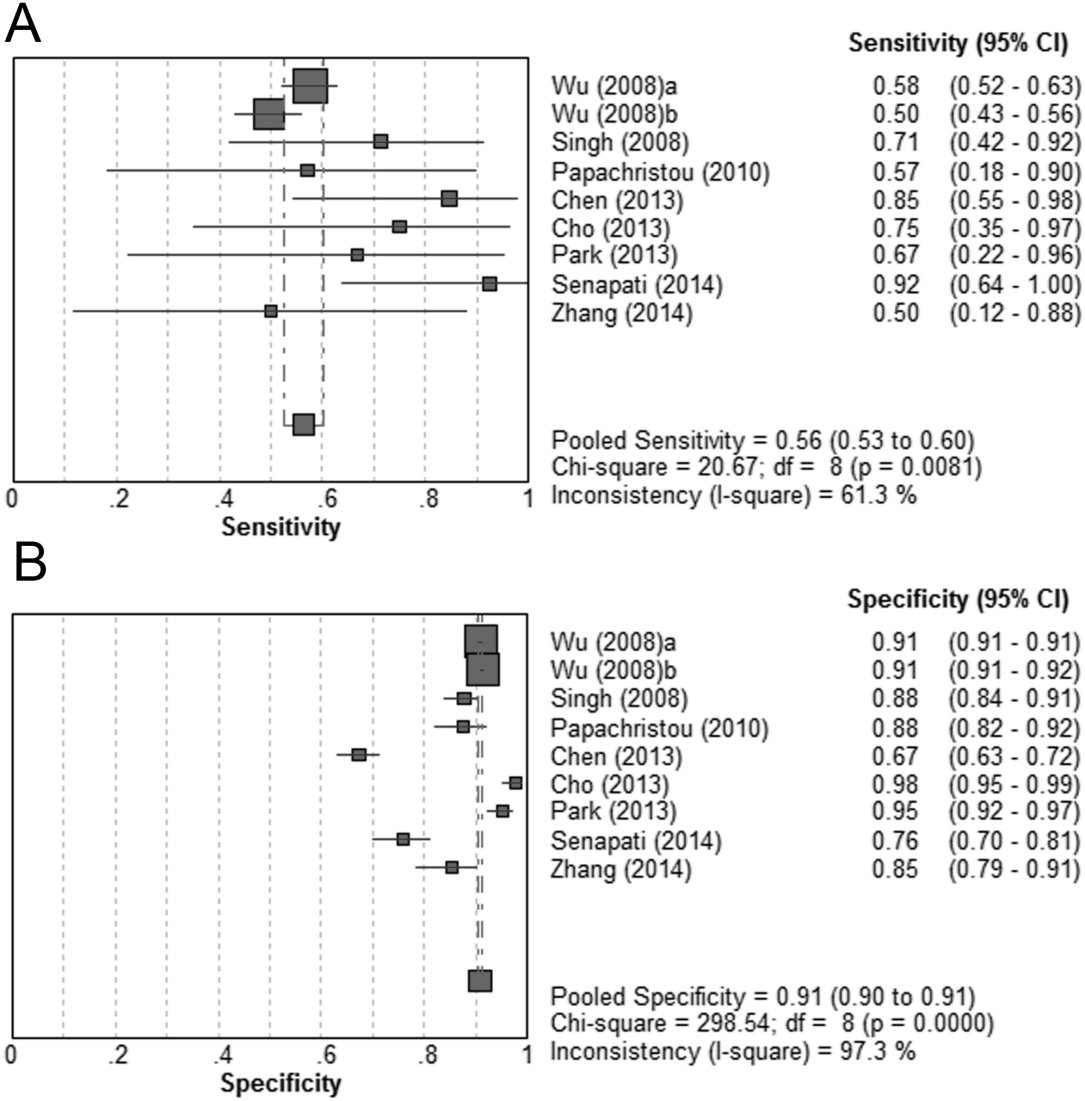
Pooled sensitivity and specificity for BISAP score ≥3 in predicting mortality. (A) Sensitivity; (B) specificity.

**Fig 3:**
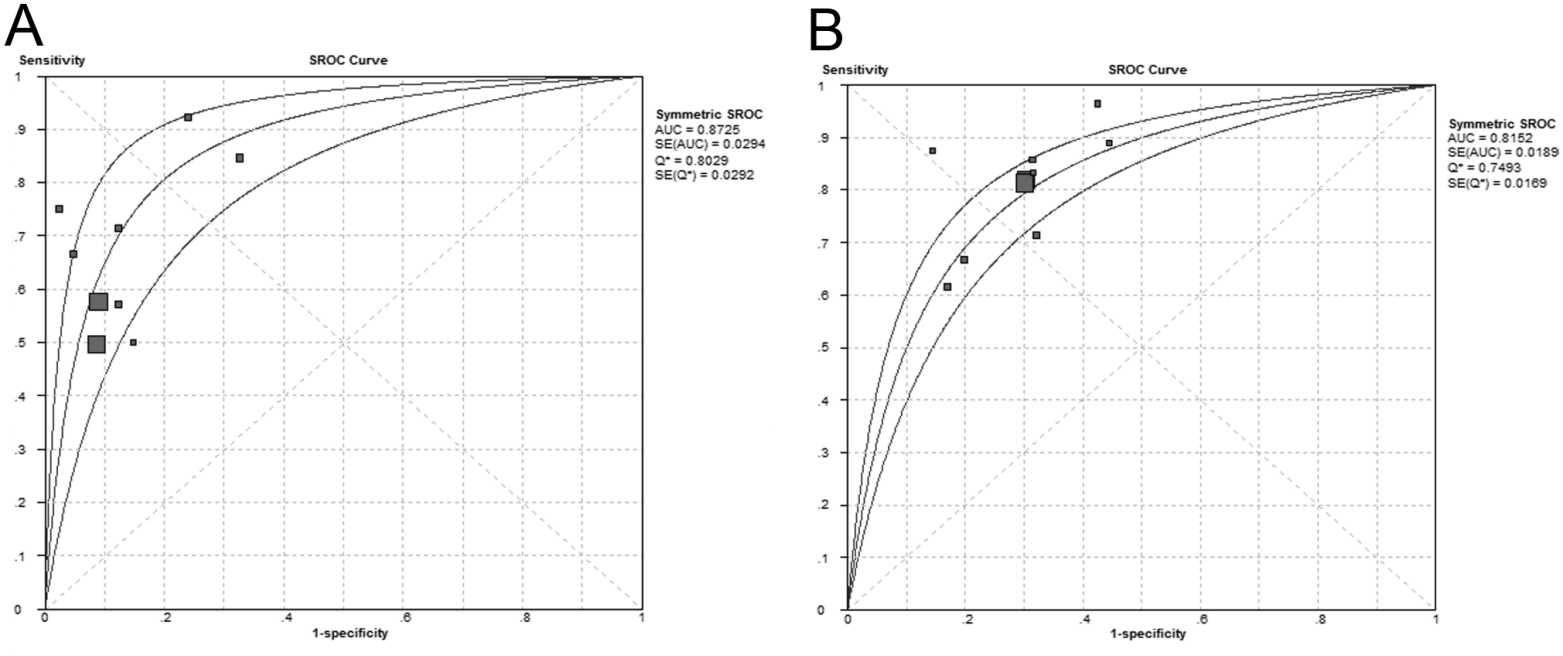
Summary of receiver operating characteristic curves of BISAP score for predicting mortality. (A) BISAP ≥3; (B) BISAP ≥2.

Nine cohorts from 8 studies were identified for the BISAP score at a cut-off of ≥3 [9,21,23–26,28,29]. Patients with a BISAP score ≥3 significantly had an higher likelihood of mortality (DOR = 13.72; 95% CI, 9.82–19.18; P < 0.05). No significant heterogeneity was revealed (P = 0.10; I^2^ = 39.9%). The pooled sensitivity was 56% (95% CI, 53%-60%), and the pooled specificity was 91% (95% CI, 90%-91%). (Fig 2A and 2B) The summary PLR and NLR were 5.65 (95% CI, 4.23–7.55) and 0.48 (95% CI, 0.41–0.56), respectively. The SROC curve yielded an AUC of 0.87 (Fig 3A). (Table 2)

**Table 2:** Pooled results of BISAP score, Ranson score, and APACHEII score for the prediction of clinical outcomes.

Clinical outcome	Score, cut-off value	Cohort, n	SEN (95% CI)	SPE (95% CI)	PLR (95% CI)	NLR (95% CI)	DOR (95% CI)	AUC (SE)	Q (SE)
Mortality	BISAP, 3	9	0.56 (0.53–0.60)	0.91 (0.90–0.91)	5.65 (4.23–7.55)	0.48 (0.41–0.56)	13.72 (9.82–19.18)	0.87 (0.03)	0.80 (0.03)
	BISAP, 2	10	0.81 (0.78–0.84)	0.70 (0.70–0.71)	2.72 (2.44–3.04)	0.27 (0.23–0.32)	10.18 (8.33–12.45)	0.82 (0.02)	0.75 (0.02)
	Ranson, 3	4	0.93 (0.78–0.99)	0.69 (0.65–0.73)	3.27 (2.03–5.26)	0.15 (0.05–0.45)	23.44 (6.91–79.47)	0.92 (0.05)	0.85 (0.06)
	APACHE II, 8	3	0.95 (0.77–1.00)	0.68 (0.63–0.73)	2.74 (2.26–3.33)	0.15 (0.04–0.54)	20.92 (4.72–92.67)	0.83 (0.16)	0.76 (0.14)
SAP	BISAP, 3	6	0.51 (0.43–0.60)	0.91 (0.89–0.92)	7.23 (4.21–12.42)	0.56 (0.44–0.71)	18.08 (8.27–39.55)	0.87 (0.06)	0.80 (0.06)
	BISAP, 2	5	0.63 (0.55–0.70)	0.82 (0.79–0.84)	3.51 (2.24–5.52)	0.44 (0.27–0.73)	8.45 (3.46–20.65)	0.88 (0.04)	0.81 (0.04)
	Ranson, 3	6	0.66 (0.59–0.72)	0.78 (0.76–0.81)	4.05 (2.26–7.27)	0.36 (0.22–0.60)	13.35 (4.53–39.36)	0.83 (0.08)	0.76 (0.07)
	APACHE II, 8	5	0.83 (0.77–0.88)	0.59 (0.56–0.63)	2.54 (1.72–3.73)	0.26 (0.18–0.40S)	10.77 (6.80–17.07)	0.82 (0.03)	0.75 (0.03)

AUC, area under curve; DOR, diagnostic odds ratio; NLR, negative likelihood ratio; PNec, pancreatic necrosis; PLR, positive likelihood ratio; SE, standard error; SEN, sensitivity; SPN, specificity.

No publication bias was shown by the funnel plot or detected by the Deek’s test (P = 0.23). Sensitivity analyses were conducted by excluding studies one at a time to determine if a particular study was responsible for the heterogeneity. When excluding the study by Wu et al. [9], which weighed the largest sample size, no substantial difference was detected for the diagnostic performance (DOR = 19.68; 95% CI, 9.47–40.89; P < 0.05) or heterogeneity (P = 0.20; I^2^ = 30.5%). Only when excluding the study by Cho et al. [23], no heterogeneity was detected (P = 0.43; I^2^ = 0). Subgroup analyses were conducted in terms of study design, sample size, main etiology, location, and prevalence of SAP. Notably, studies with a sample size below 300 produced DOR estimates nearly twofold higher than studies with a sample size over 300. Studies with main etiology of biliary stone showed DOR estimates that were about 2.5 folds higher than the studies with main etiology of alcohol. The Asian studies showed DOR estimates that were about twofold higher than the American studies. Studies with a prevalence of SAP below 10% produced DOR estimates that were about three times higher than studies with a prevalence exceeding 10%. (Table 3) In the univariate meta-regression analyses, no statistical significance was revealed for study design, sample size, main etiology, or location. However, the prevalence of SAP was likely to contribute to the heterogeneity between studies (P = 0.08).

**Table 3:** Subgroup analyses for cohorts assessing the predictive value of BISAP ≥3 for mortality.

Subgroups	Cohort, n	DOR (95% CI)	AUC	Cochrane-Q	P value	I^2^
Study design						
Prospective	3	16.79 (7.08–39.82)	0.90	1.23	0.54	0
Retrospective	6	13.59 (9.07–20.37)	0.83	11.75	0.04	57.4%
Sample size						
≥ 300	5	12.49 (10.02–15.58)	0.87	4.89	0.30	18.1%
< 300	4	21.21 (5.36–84.00)	0.89	7.41	0.06	59.5%
Main etiology						
Biliary	5	12.29 (10.41–14.51)	0.87	3.30	0.51	0
Alcohol	4	31.25 (8.29–117.81)	0.91	6.45	0.09	53.5
Location						
USA	4	12.26 (10.16–14.79)	0.93	3.29	0.35	8.8%
Asia	5	24.68 (8.35–72.99)	0.89	7.72	0.10	48.2%
SAP%						
≥ 10%	4	11.97 (5.32–26.94)	0.83	2.66	0.45	0
< 10%	3	37.85 (11.61–123.35)	0.92	3.16	0.21	36.8%

**Fig 4:**
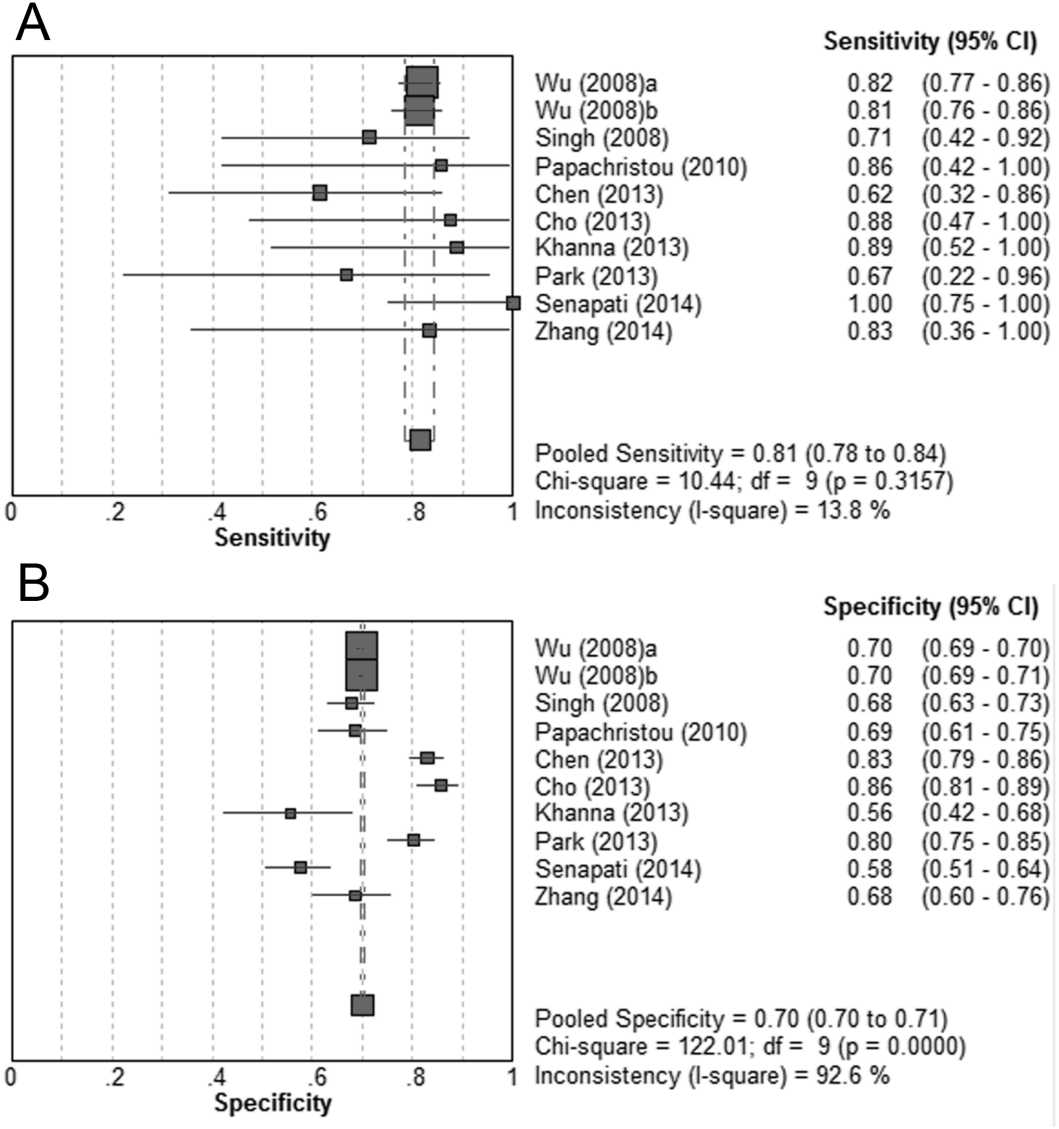
Pooled sensitivity and specificity for BISAP score ≥2 in predicting mortality. (A) Sensitivity; (B) specificity.

Further, we performed sensitivity analyses by assessing the BISAP score at a cut-off of ≥2. Data could be obtained or calculated in 10 cohorts from 9 studies [9,21,23–29]. Patients with a BISAP score ≥2 had significantly increased mortality than those with a BISAP score <2. No evidence of heterogeneity was revealed (DOR = 10.18; 95% CI, 8.33–12.45; P < 0.05; I^2^ = 0%). Compared with BISAP ≥3, the sensitivity increased and the specificity decreased for BISAP at a cut-off of ≥2. The pooled sensitivity was 81% (95% CI, 78%-84%), and the specificity was 70% (95% CI, 70%-71%). (Fig 4A and 4B) The summary PLR was 2.72 (95% CI, 2.44–3.04), and the pooled and NLR was 0.27 (95% CI, 0.23–0.32). The SROC curve revealed an AUC of 0.82 (Fig 3B). (Table 2)

#### SAP

**Fig 5:**
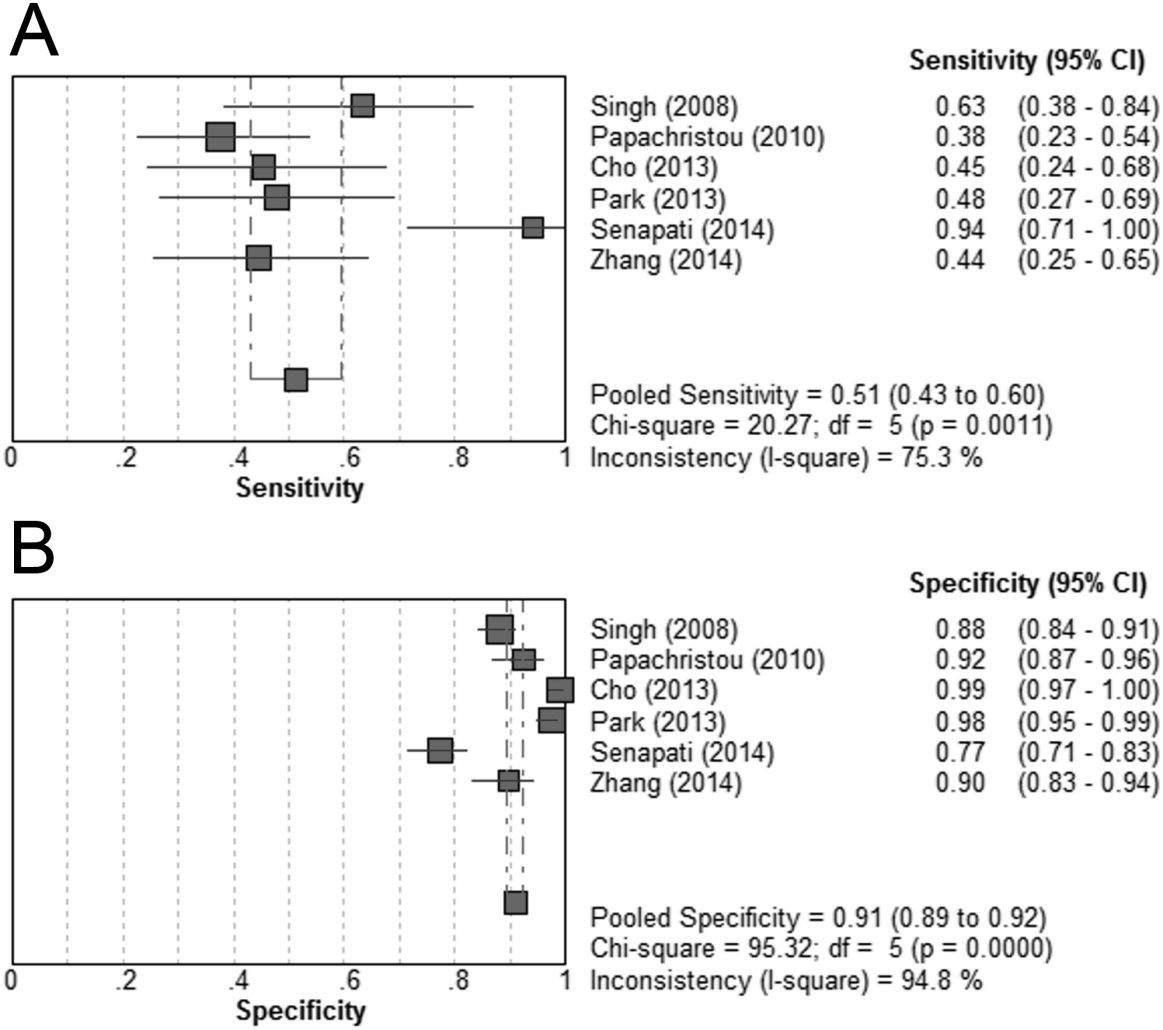
Pooled sensitivity and specificity for BISAP score ≥3 in predicting severe acute pancreatitis. (A) Sensitivity; (B) specificity.

Data relating to a BISAP score of ≥3 could be extracted or calculated from 6 studies [21,23,24,26,28,29]. The BISAP score ≥3 was significantly associated with increased risk of SAP (DOR = 18.08; 95% CI, 8.27–39.55; P < 0.05; I^2^ = 64.2%). The pooled sensitivity was 51% (43%-60%), and the pooled specificity was 91% (89%-92%). (Fig 5) The summary PLR and NLR were 7.23 (4.21–12.42) and 0.56 (0.44–0.71), respectively. The SROC curve showed an AUC of 0.87. (Table 2)

In sensitivity analyses by excluding studies one by one, no single study fully explained the high heterogeneity. Subgroup analyses were performed in terms of study design, sample size, location, and prevalence of SAP. Notably, retrospective studies showed DOR estimates that were about twofold of the prospective studies. Studies of Asian population showed DOR estimates that were about three times higher than studies of American population, which was the same for results of etiology subgroup comparison. Studies with a prevalence of SAP below 10% produced DOR estimates that were about three times higher than studies with a prevalence exceeding 10%. (Table 4) In univariate meta-regression analyses, no statistical significance was revealed for study design, sample size, location, or the prevalence of SAP.

**Table 4:** Subgroup analyses for cohorts assessing the predictive value of BISAP ≥3 for severe acute pancreatitis defined by the latest 2012 Atlanta classification.

Subgroups	Cohort, n	DOR (95% CI)	AUC	Cochrane-Q	P value	I^2^
Study design						
Prospective	3	12.45 (5.08–30.53)	0.90	3.51	0.17	43.1
Retrospective	3	25.09 (6.02–104.55)	0.40	9.32	0.01	78.5
Sample size						
≥ 300	2	20.61 (7.41–57.29)	-	1.91	0.17	47.7
< 300	4	17.84 (5.50–57.86)	0.89	11.09	0.01	73
Main etiology						
Biliary	2	9.36 (4.85–18.09)	-	0.67	0.41	0
Alcohol	4	28.47 (8.70–93.10)	0.91	9.92	0.02	69.8
Location						
USA	2	9.36 (4.85–18.09)	-	0.67	0.41	0
Asia	4	28.47 (8.70–93.10)	0.91	9.92	0.02	69.8
SAP%						
≥ 10%	3	11.74 (4.38–31.46)	0.31	6.09	0.05	67.1
< 10%	3	32.58 (9.12–116.38)	0.91	4.80	0.09	58.3

Sensitivity analyses were performed by evaluating the BISAP score at a cut-off of ≥2. Data could be obtained or calculated in 5 cohorts from 4 studies [22–24,26]. Patients with a BISAP score of ≥2 were at significantly increased risk for SAP (DOR = 8.45; 95% CI, 3.46–20.65; P < 0.05; I^2^ = 80.5%). Compared with the cut-off of ≥3, the sensitivity increased and the specificity decreased for the cut-off of ≥2. The pooled sensitivity was 63% (55%-70%), and the specificity was 82% (79%-84%). The summary PLR was 3.51 (95% CI, 2.24–5.52), and the NLR was 0.44 (95% CI, 0.27–0.73). The SROC curve yielded an AUC of 0.88. (Table 2)

### Results of Ranson Score

#### Mortality

Four studies were available for the Ranson score at a cut-off of ≥3 [23,26–28]. The Ranson score of ≥3 was significantly associated with increased mortality in patients with AP (DOR = 23.44; 95% CI, 6.91–79.47; P < 0.05). No significant heterogeneity was revealed (P = 0.75; I2 = 0). The pooled sensitivity was 93% (95% CI, 78%-99%), and the specificity was 69% (95% CI, 65%-73%). The summary PLR and NLR were 3.27 (95% CI, 2.03–5.26) and 0.15 (95% CI, 0.05–0.45), respectively. The SROC curve yielded an AUC of 0.92. (Table 2)

#### SAP

Six cohorts from 5 studies were available for the Ranson score at a cut-off of ≥3 [22,23,26–28]. The Ranson score of ≥3 was significantly associated with increased risk of SAP (DOR = 13.35; 95% CI, 4.53–39.36; P < 0.05), with significant heterogeneity (P < 0.01; I^2^ = 87.3%). The pooled sensitivity was 66% (95% CI, 59%-72%), and the specificity was 78% (95% CI, 76%-81%). The summary PLR and NLR were 4.05 (95% CI, 2.26–7.27) and 0.36 (95% CI, 0.22–0.60), respectively. The SROC curve revealed an AUC of 0.83. (Table 2)

### Results of APACHEII Score

#### Mortality

Three studies were available for the APACHEII score at a cut-off of ≥8 [26–28]. The APACHEII score ≥8 was significantly associated with increased mortality in patients with AP (DOR = 20.92; 95% CI, 4.72–92.67; P < 0.05). No significant heterogeneity was revealed (P = 0.86; I2 = 0). The pooled sensitivity was 95% (95% CI, 77%-100%), and the specificity was 68% (95% CI, 63%-73%). The summary PLR and NLR were 2.74 (95% CI, 2.26–3.33) and 0.15 (95% CI, 0.04–0.54), respectively. The SROC curve showed an AUC of 0.83. (Table 2)

#### SAP

Five cohorts from 4 studies were selected for the APACHEII score at a cut-off of ≥8 [22,26–28]. Patients with a APACHEII score of ≥8 had significantly increased risk of SAP (DOR = 10.77; 95% CI, 6.80–17.07; P < 0.05). No significant heterogeneity was detected (P < 0.37; I^2^ = 5.7%). The pooled sensitivity was 83% (95% CI, 77%-88%), and the specificity was 59% (95% CI, 56%-63%). The summary PLR and NLR were 2.54 (95% CI, 1.72–3.73) and 0.26 (95% CI, 0.18–0.40), respectively. The SROC curve yielded an AUC of 0.82. (Table 2)

## Discussion

The present study focused on the predictive value of BISAP score for assessing clinical outcomes of AP. Our pooled results showed that the BISAP score at a cut-off of ≥3 had a moderate sensitivity and a high specificity for predicting mortality and SAP. In comparison, at a cut-off of ≥2, the sensitivity increased whereas the specificity decreased for both outcomes. When calculating the likelihood ratios for BISAP score at a threshold of 3, PLRs were above 5 for both outcomes, suggesting that a BISAP score of ≥3 did well in predicting mortality and severity of AP. This is helpful that patients with SAP will be put on monitored beds early. However, the NLRs exceeded 0.2 for these outcomes at any cut-off, which indicated that a low BISAP score was not robust enough to predict patients at low risk for death or SAP. Thus, many patients with mild disease may be falsely be labeled as having mild disease when later they will develop SAP.

Over years, the Ranson criteria and APACHEIIsystem have been well-established in the assessment of patients with AP. However, both of them have significant weaknesses. The Ranson criteria requires 48 hours to complete, which will miss the potentially valuable early treatment. The APACHEIIsystem is a generic score for all critically ill patients. It requires the collection of many parameters, which may not be available outside the ICU, and some parameters may be irrelevant to the prognosis [30]. By contrast, the BISAP score is simpler to calculate and only uses routine clinical data within 24 hour of presentation.

In our meta-analysis, compared with the BISAP score, the Ranson criteria and APACHEIIscore both showed higher sensitivity and lower specificity for predicting mortality and SAP. Especially, the sensitivity was remarkably high when employing the two conventional scoring systems to predict mortality. The NLRs came up to 0.15 for both Ranson criteria and APACHEIIscore, indicating that a low score of both scoring systems was reliable to identify the patients at low risk for death.

In the subgroups of sample size < 300, main etiology of alcohol, Asian population, and SAP < 10%, a BISAP score of ≥3 appeared to be more effective in predicting mortality and SAP. However, in meta-regression analyses, only SAP < 10% was weakly suggestive as a source of heterogeneity. For studies of smaller sample size or lower proportion of SAP, the effect sizes may be overestimated, thus causing higher DOR. The American studies all enrolled patients mainly caused by gallstones, and the Asian studies predominantly included patients with alcohol-induced pancreatitis. In a previous study, three prognostic indices, including clinical assessment, multiple laboratory criteria, and peritoneal lavage, have been compared for the predictive value of severity of AP [31]. Similar with our findings, each of the indices was more accurate in diagnosing the severity of alcohol than gallstone pancreatitis. Further studies were warranted to clarify the influence of etiology on the predictive value of scoring systems.

There were several strengths to the current study. We included 12 cohorts from 10 studies, encompassing 38985 patients. The broad sample of patients from which the statistical estimates were yielded showed a high degree of external validity of our findings. SAP was defined by the latest updated 2012 Atlanta classification. Results of different cut-offs was investigated separately. Subgroup analyses and meta-regression analyses were conducted to thoroughly explore the sources of heterogeneity. Additionally, the predictive accuracy of BISAP score was compared with the traditional Ranson criteria and APACHEIIscore.

We were aware of the limitations of this meta-analysis. Firstly, as only articles written in English were included, we may miss relevant studies published in non-English language journals. Articles with statistically significant data were more likely to appear in English language journals. Although publication bias was not detected, it was limited by the small number of studies. Secondly, statistical heterogeneity was noted between studies, especially when assessing the outcome of SAP. As only 12 cohorts from 10 studies were included into the meta-analysis, compounded by the small sample sizes of several studies, it may be insufficient to yield robust results through subgroup analyses or meta-regression analyses. Only half of the cohorts were prospectively designed. Retrospective studies may limit the comparison of BISAP score, Ranson criteria and APACHEIIscore. Besides, we could not obtain sufficient data for the transferred patients, such as SIRS and the presence/absence of pleural effusion on imaging. Although all studies calculated BISAP score within 24 hours after admission, no study showed the BISAP score on admission. The reports of laboratory tests or chest X ray could hardly be obtained immediately on admission in most hospitals, which may delay the calculation of BISAP score. Only one study compared BISAP score with blood urea nitrogen or SIRS alone [22], which limited the systematical comparison between BISAP score and single parameters. In addition, considerable clinical variations between studies may influence the predictive accuracy of BISAP score. For example, the commonly reported prevalence of SAP in literature was 10% to 20%, whereas several studies reported a prevalence below 10% or over 20%. Most studies included patients with AP of various etiologies. Our subgroup analyses also demonstrated the discrepancies when evaluating these confounding factors.

This meta-analysis was the first attempt to systematically examine the performance of BISAP score for predicting the clinical outcomes of patients with AP. Our results confirmed that BISAP score was a useful tool for predicting mortality and SAP defined by the latest 2012 Atlanta classification. Compared with the Ranson criteria and APACHEIIscore, the BISAP score showed higher specificity and lower sensitivity for mortality and SAP. A BISAP score of ≥3 seemed to be reliable to identify the high-risk AP patients. Further well-designed prospective studies were warranted to investigate more convenient scoring systems with both high specificity and sensitivity.

## Supporting Information

S1 Checklist PRISMA Checklist (DOC)

S1 Flow Diagram PRISMA 2009 Flow Diagram (DOC)

S1 Table Results of quality assessment by the revised 7-item assessment tool. (DOCX)

S1 Text Full-text excluded articles.doc. (DOCX)
