# Correction: The Value of BISAP Score for Predicting Mortality and Severity in Acute Pancreatitis: A Systematic Review and Meta-Analysis

**DOI:** 10.1371/journal.pone.0142025

**Published:** 2015-10-29

**Authors:** Wei Gao, Hong-Xia Yang, Cheng-En Ma

There is an error in the affiliation for all of the authors. The affiliation should be: Department of Intensive Care Unit, the Second Hospital of Shandong University, Jinan, 250033, China.

## References

[1] Gao W, Yang H-X, Ma C-E (2015) The Value of BISAP Score for Predicting Mortality and Severity in Acute Pancreatitis: A Systematic Review and Meta-Analysis. PLoS ONE 10(6): e0130412 doi: 10.1371/journal.pone.0130412 26091293 10.1371/journal.pone.0130412 PMC4474919

