# Supplementary material for: The Value of BISAP Score for Predicting Mortality and Severity in Acute Pancreatitis: A Systematic Review and Meta-Analysis
Source: PLoS One. 2015 Jun 19;10(6):e0130412. doi: 10.1371/journal.pone.0130412 (PMC4474919; doi:10.1371/journal.pone.0130412)
Supplement: S1 Flow Diagram — (DOC) [file pone.0130412.s002.doc]

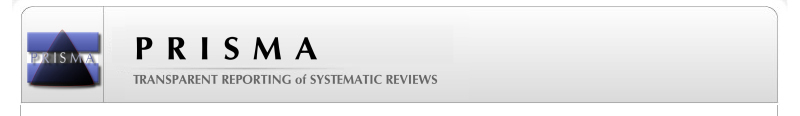
**PRISMA 2009 Flow Diagram**

Records identified through database searching:

Pubmed (25)

Embase (19)

Cochrane Library (0)

(n = 44)

**Screening**

**Included**

**Eligibility**

**Identification**

Records screened

(n = 29)

15 duplicate records were excluded

Full-text articles assessed for eligibility
(n = 23)

Studies included in qualitative synthesis
(n = 13)

Studies included in quantitative synthesis (meta-analysis)
(n = 10)

6 reviews were excluded

10 studies reporting insufficient data on BISAP

3 studies were excluded:

2 studies reporting SAP defined by 1992 Atlanta classification

1 study of the same cohort
