# Supplementary material for: The Value of BISAP Score for Predicting Mortality and Severity in Acute Pancreatitis: A Systematic Review and Meta-Analysis
Source: PLoS One. 2015 Jun 19;10(6):e0130412. doi: 10.1371/journal.pone.0130412 (PMC4474919; doi:10.1371/journal.pone.0130412)
Supplement: S1 Table — (DOCX) [file pone.0130412.s003.docx]

| Author (year) | Selection of patients in an unbiased fashion | Representative of a wide spectrum of AP severity | BISAP assessed blinded to outcome | Outcome assessed blinded to BISAP score | Accurate definition of outcomes (especially SAP) | Availability of the same clinical data | Adequate follow-up |
| --- | --- | --- | --- | --- | --- | --- | --- |
| Wu (2008) | Yes | Yes | Not clear | Not clear | Yes | Yes | Yes |
| Singh (2008) | Yes | Yes | Yes | Not clear | Yes | Yes | Yes |
| Papachristou (2010) | Yes | Yes | Yes | Not clear | Yes | Yes | Yes |
| Mounzer (2012) | Yes | Yes | Yes | Not clear | Yes | Yes | Yes |
| Chen (2013) | Yes | Yes | Not clear | Not clear | Yes | Yes | Yes |
| Cho (2013) | Yes | Yes | Not clear | Not clear | Yes | Yes | Yes |
| Khanna (2013) | Yes | Yes | Yes | Not clear | Yes | Yes | Yes |
| Park (2013) | Yes | Yes | Not clear | Not clear | Yes | Yes | Yes |
| Senapati (2014) | Yes | Yes | Yes | Not clear | Yes | No | Yes |
| Zhang (2014) | Yes | Yes | Not clear | Not clear | Yes | Yes | Yes |
