# Supplementary material for: The Value of BISAP Score for Predicting Mortality and Severity in Acute Pancreatitis: A Systematic Review and Meta-Analysis
Source: PLoS One. 2015 Jun 19;10(6):e0130412. doi: 10.1371/journal.pone.0130412 (PMC4474919; doi:10.1371/journal.pone.0130412)
Supplement: S1 Text — (DOCX) [file pone.0130412.s004.docx]

**S1 Text. Thirteen full-text excluded articles.**

**Two studies reported SAP defined by the 1992 Atlanta classification**

1. Bezmarevic M, Kostic Z, Jovanovic M, Mickovic S, Mirkovic D, et al. (2012) Procalcitonin and BISAP score versus C-reactive protein and APACHE II score in early assessment of severity and outcome of acute pancreatitis. Vojnosanitetski Pregled 69: 425-431.

2. Kim BG, Noh MH, Ryu CH, Nam HS, Woo SM, et al. (2013) A comparison of the BISAP score and serum procalcitonin for predicting the severity of acute pancreatitis. Korean Journal of Internal Medicine 28: 322-329.

**One study of the same cohort as Singh et al. 2008**

1. Bollen TL, Singh VK, Maurer R, Repas K, Van Es HW, et al. (2012) A comparative evaluation of radiologic and clinical scoring systems in the early prediction of severity in acute pancreatitis. American Journal of Gastroenterology 107: 612-619.

**Ten studies reported insufficient data on BISAP**

1.Wang S, Feng X, Li S, Liu C, Xu B, et al. (2014) The ability of current scoring systems in differentiating transient and persistent organ failure in patients with acute pancreatitis. J Crit Care 29: 693.e697-611.

2. Francisco M, Valentin F, Cubiella J, Fernandez-Seara J (2013) Factors related to length of hospital admission in mild interstitial acute pancreatitis. Revista Espanola de Enfermedades Digestivas 105: 84-92.

3. Jin Y, Lin CJ, Dong LM, Chen MJ, Zhou Q, et al. (2013) Clinical significance of melatonin concentrations in predicting the severity of acute pancreatitis. World Journal of Gastroenterology 19: 4066-4071.

4. Kerdsirichairat T, Attam R, Arain M, Bakman Y, Radosevich D, et al. (2014) Urgent ERCP with pancreatic stent placement or replacement for salvage of post-ERCP pancreatitis. Endoscopy 46: 1085-1091.

5. Cardoso FS, Ricardo L, Gondar P, Deus JR, Horta D (2015) C-reactive protein may influence decisively the prescription of prophylactic antibiotics in acute pancreatitis: a population-based cohort study. Pancreas 44: 404-408.

6. Wu BU, Conwell DL, Singh VK, Repas K, Maurer R, et al. (2010) Early hemoconcentration is associated with pancreatic necrosis only among transferred patients. Pancreas 39: 572-576.

7. Singh VK, Bollen TL, Wu BU, Repas K, Maurer R, et al. (2011) An assessment of the severity of interstitial pancreatitis. Clin Gastroenterol Hepatol 9: 1098-1103.

8. Francisco M, Valentin F, Cubiella J, Alves MT, Garcia MJ, et al. (2012) Factors associated with intolerance after refeeding in mild acute pancreatitis. Pancreas 41: 1325-1330.

9. Karsenti D, Viguier J, Bourlier P, d'alteroche L, Barbieux JP, et al. (2003) Enteral nutrition during acute pancreatitis: feasibility study of a self-propeeling spiral distal end jejunal tube. Gastroenterol Clin Biol 27: 614-617.

10. Talukdar R, Bhattacharrya A, Rao B, Sharma M, Nageshwar Reddy D (2014) Clinical utility of the revised Atlanta classification of acute pancreatitis in a prospective cohort: have all loose ends been tied? Pancreatology 14: 257-262.
